# A comparative study of prokaryotic diversity and physicochemical characteristics of Devils Hole and the Ash Meadows Fish Conservation Facility, a constructed analog

**DOI:** 10.1371/journal.pone.0194404

**Published:** 2018-03-15

**Authors:** Joshua D. Sackett, Desiree C. Huerta, Brittany R. Kruger, Scott D. Hamilton-Brehm, Duane P. Moser

**Affiliations:** 1 Division of Earth and Ecosystems Sciences, Desert Research Institute, Las Vegas, Nevada, United States of America; 2 School of Life Sciences, University of Nevada, Las Vegas, Nevada, United States of America; 3 Division of Hydrologic Sciences, Desert Research Institute, Las Vegas, Nevada, United States of America; Brandenburgische Technische Universitat Cottbus-Senftenberg, GERMANY

## Abstract

Devils Hole is the sole natural habitat of the critically endangered Devils Hole pupfish (*Cyprinodon diabolis*). To establish a backup population, the Ash Meadows Fish Conservation Facility (AMFCF), a full-scale replica of the uppermost 6.7 m of Devils Hole, was constructed by management agencies in the mid-2010s. Despite rigorous efforts to mimic the bathymetric and physical details of the Devils Hole environment, the biogeochemistry and microbiology of the AMFCF refuge tank remain largely unaddressed. We evaluated water physicochemistry and employed Illumina DNA sequencing of 16S rRNA gene libraries to evaluate planktonic and benthic bacterial and archaeal community composition within their respective physicochemical contexts in Devils Hole and AMFCF on the same day. Major ion concentrations were consistent between the two systems, but water temperature and dissolved oxygen dynamics differed. Bioavailable nitrogen (primarily nitrate) was 5x lower in AMFCF. Devils Hole and AMFCF nitrogen:phosphorus molar ratios were 107:1 and 22:1, indicative of different nutrient control mechanisms. Both sites are microbiologically diverse, with over 40 prokaryotic phyla represented at each, with 37 shared between them and nearly than half deriving from candidate divisions. The abundance and composition of predicted photosynthetic primary producers (Cyanobacteria) was markedly different between sites: Devils Hole planktonic and sediment communities were dominated by *Oscillatoria spp*. (13.2% mean relative abundance), which proved virtually undetectable in AMFCF. Conversely, AMFCF was dominated by a predicted heterotroph from the Verrucomicrobiaceae family (31.7%); which was comparatively rare (<2.4%) in Devils Hole. We propose that the paucity of bioavailable nitrogen in AMFCF, perhaps resulting from physical isolation from allochthonous environmental inputs, is reflected in the microbial assemblage disparity, influences biogeochemical cycling of other dissolved constituents, and may ultimately impact survivorship and recruitment of refuge populations of the Devils Hole pupfish.

## Introduction

The role of microorganisms in elemental and nutrient cycling, as direct food resources for higher organisms, and in regulating overall system productivity is an often overlooked aspect of natural and constructed aquatic systems, particularly in conservation efforts of endangered fish species [1, 2]. The abundance and distribution of microorganisms is affected by physical parameters (e.g.: temperature, pH, light) and aqueous chemistry gradients (e.g.: dissolved oxygen, nutrient, carbon, and trace element concentrations) [3, 4]. Changes in the distribution and composition of microbial assemblages in ecosystems that support endangered species, or the inability to replicate the lower trophic structure and aqueous physicochemistry in constructed refuges or mesocosms, can affect the survivorship of higher order organisms. This is due to alteration of dissolved nutrient availability and distribution, reduction or destruction of habitat, and/or loss of food sources for higher-order organisms [5]. These changes, which propagate through the aquatic food web, are consequential to endangered endemic fish populations within their respective habitats.

Endemic fishes of the arid southwestern United States are among the planet’s most imperiled species. The desert aquatic ecosystems that support populations of native fishes are increasingly threatened by a range of interrelated factors, including the influence of invasive and introduced species [6], groundwater diversion [7], and climate change [8, 9]. Devils Hole, a cavernous limnocrene located in a disjunct portion of Death Valley National Park in the Mojave Desert, Nevada, USA [1], is one such vulnerable and threatened desert aquatic ecosystem. Devils Hole supports a relatively simple food web, composed of floating biofilms dominated by filamentous Cyanobacteria (*Oscillatoria* and *Plectonema*), a eukaryotic alga (*Spirogyra*), and a heterotrophic Gammaproteobacterium (*Beggiatoa*) [10], fewer than 15 recognized invertebrate species [11], and a single vertebrate: a critically endangered, endemic cyprinodont fish (*Cyprinodon diabolis*, the Devils Hole pupfish) [12–14]. The small (<30mm in length), energetic, iridescent blue Devils Hole pupfish survive on a diet composed of diatoms, cyanobacteria, filamentous green algae, and macroinvertebrates [15, 16]. Often regarded as the most geographically restricted vertebrate on the planet [1, 17], a combination of extremely low fecundity, unpredictable survivorship, and a short 12-14-month lifecycle renders the population intrinsically vulnerable to environmental and hydrologic disturbances. Correspondingly, the Devils Hole pupfish and its habitat have been the focus of extensive litigation (United States vs. Cappaert, 1974; 1978) [1], including the first test of the US Endangered Species Act (Cappaert vs. the United States, 1976), public engagement, and monitoring/conservation efforts since the 1960s.

Local groundwater extraction in the 1960s and early 1970s caused the water level in Devils Hole to fall, exposing the shallow rock shelf critical for Devils Hole pupfish feeding and spawning, and thereby driving a decline in the pupfish population (annual maxima $\bar{\text{x}}=296$ individuals) during the lowest water years of 1972–1976. Following a court-ordered curtailment of groundwater withdrawal, however, the pupfish population partially rebounded in the 1980s and 1990s (annual maxima $\bar{\text{x}}=482$ individuals [14]); only to subsequently decline again to an historic low of just 35 individuals in the spring of 2013 [8], which coincided with an observed decrease in *Spirogyra* (eukaryotic algae) and cyanobacterial biofilm biomass [10]. In an effort to prevent extinction of the species, three artificial refuges were constructed over the years: Hoover Dam, School Springs, and Point of Rocks (completed in 1972, 1973, and 1990, respectively). Following some initial successes, all ultimately failed primarily due to fluctuations in water supply and temperature (engineering failures) and accumulation of sediment and debris [18–20]. Most recently (mid 2000s), interspecies hybridization (*C*. *diabolis* × *C*. *nevadensis mionectes*, the Ash Meadows Amargosa pupfish) and subsequent phenotypic divergences were observed at the Point of Rocks Refuge [18], making release of its fish back into Devils Hole untenable [19–21].

Attempts to cultivate Devils Hole pupfish in laboratory settings have also been challenging. In 1995, for example, Deacon et al. showed that hatch rates were greatly reduced in laboratory aquaria (4%) vs. Devils Hole (12%) [22]. More recently, Feuerbacher and colleagues demonstrated that egg and larval infections contributed to low hatch rates and reduced larval survival of Devils Hole pupfish hybrids in constructed environments [23]. In 2013, another effort was undertaken to establish a backup population of Devils Hole pupfish, which culminated in the construction of the Ash Meadows Fish Conservation Facility (AMFCF). To a greater extent than with the earlier examples, this artificial habitat was designed to replicate the dimensions, microclimate, and aqueous chemistry of Devils Hole. Physical conditions in AMFCF, however, intentionally deviate from those in Devils Hole to encourage fish survival and reproduction through reduced thermal and respiratory stress. Specifically, water temperature at AMFCF is maintained 2–3°C lower than in Devils Hole (33.5°C ambient) and dissolved oxygen concentration is roughly double that in Devils Hole (~5 mg/L vs. 2.5 mg/L); modifications empirically shown in work conducted in the AMFCF laboratory to enhance egg production, growth, and survival of *C*. *diabolis* × *C*. *nevadensis mionectes* hybrids [24]. In spite of these amendments, minimal natural reproduction of Devils Hole pupfish has been documented in AMFCF to date and recruitment success has been limited [25].

To date, constructed refuges have focused primarily on maintaining the Devils Hole pupfish phenotype while generally ignoring physical and chemical parameters, particularly nutrient dynamics, and microbial influences. We hypothesize that the limited survivorship and recruitment success of the Devils Hole pupfish in AMFCF may reflect a failure to replicate Devils Hole nutrient balance and microbiology. As biogeochemical function of oligotrophic ecosystems is thought to be disproportionately influenced by prokaryotes [5], ecosystem sensitivity to lower trophic dysfunction could be magnified in Devils Hole and AMFCF. Thus, the ongoing lack of attention to microbial ecology could represent both a knowledge gap in our understanding of historic declines of wild Devils Hole pupfish and a factor affecting the success of aquatic refuges in general. The work presented here represents the first published survey of prokaryotic diversity at Devils Hole and the AMFCF and a first step towards the development of a baseline understanding of the microbial ecology of these sites. Here, we provide a side-by-side assessment of how effectively AMFCF replicates the extant Devils Hole environment, which in turn may provide insights for the management of Devils Hole pupfish in both environments.

## Materials and methods

### Site description

**Fig 1:**
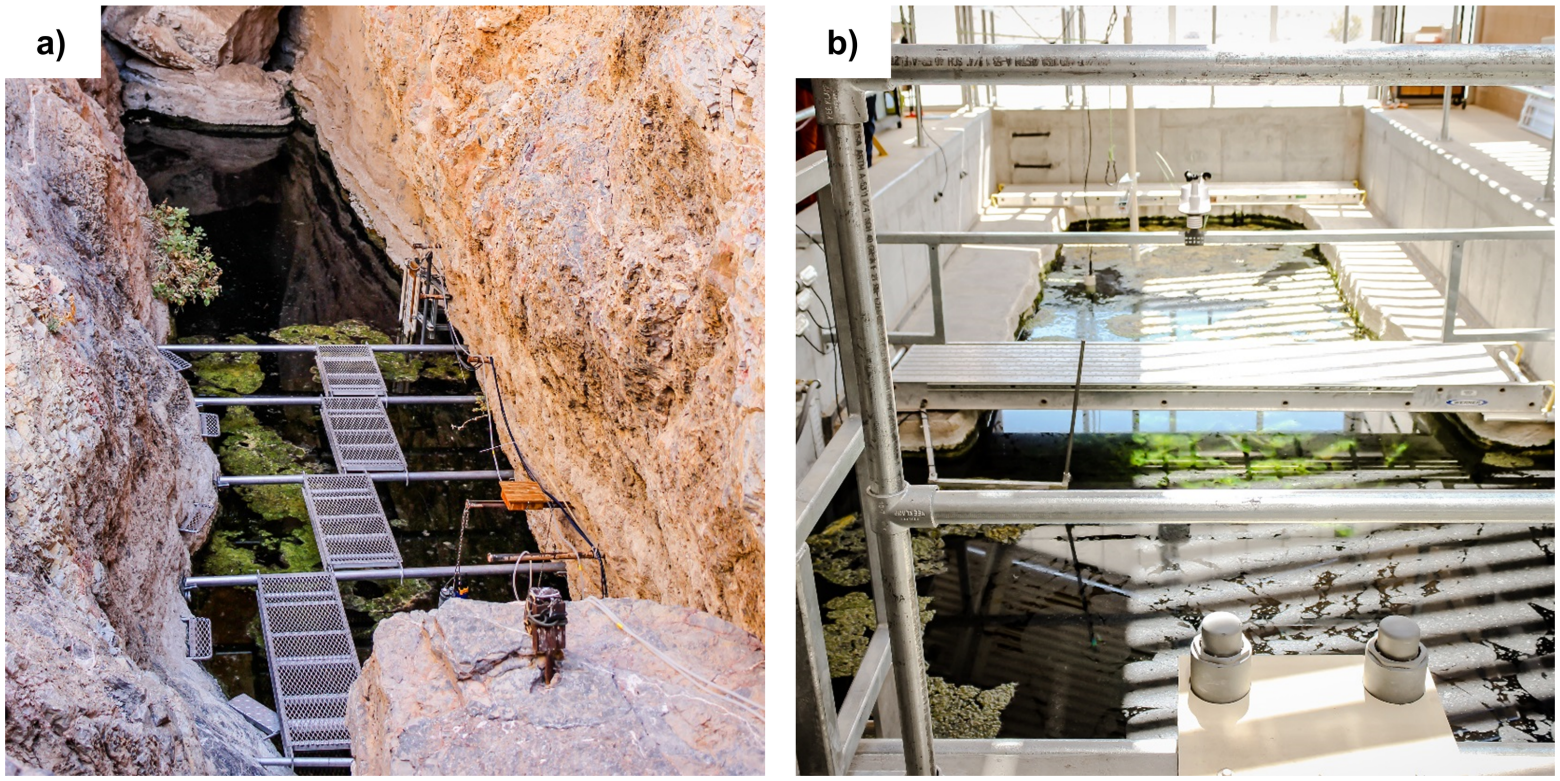
Field sites. a) The top of the Devils Hole water table viewed looking north. The shallow shelf roughly corresponds to the area below the metal walkway (temporarily installed for this work) and was partially covered with algal mats on the day of sampling. The deep pool occupies the algae-free upper half of the submerged area. b) Refuge tank at the Ash Meadows Fish Conservation Facility, a full-scale bathymetric replica of the shelf at Devils Hole, on the day of sampling. As shown, the shelf occupies the area below the concrete ledge.

Devils Hole (36°25’38”N, 116°17’28”W), located in a disjunct portion of Death Valley National Park within the Ash Meadows National Wildlife Refuge (AMNWR), Amargosa Valley, Nevada, USA, is a tectonic cave in Paleozoic carbonate rock and classified as a geothermal limnocrene (Fig 1a) [1]. The cave is narrow and elongate with near-vertical sides, and intersects local groundwater at ~15 m below land surface [15]. The water body (~3.5 x 22 m surface area) is separated into a deep pool and a shallow rock shelf. The pool has an unknown maximum depth (>152 m) and is sourced by a deep fractured rock aquifer, the Death Valley Regional Flow System [26–29] underlying AMNWR [30]. The shelf occupies ~3.5 x ~5 m at the south end of Devils Hole and is variably covered with cobble, gravel and fine sediments, deposited and rearranged periodically by meteoric runoff or seismically induced seiches [30]. This shelf exists ~30 cm below water surface and hosts seasonal algal blooms, despite receiving at most 4–5 hours of direct sunlight during the summer and only indirect illumination for ~7 months of the year [15, 18]. Most of Devils Hole pupfish feeding and spawning occurs on this shelf [31].

AMFCF (36°25’25”N, 116°18’20”W), completed in 2013 and stocked periodically with material from Devils Hole (algal mats and shelf sediment), is located 1.3 km west of Devils Hole (Fig 1b). This 415,000 L, 6.7-m-deep replica of the shallower portions of Devils Hole was constructed to establish and maintain a backup population of Devils Hole pupfish. Its shelf, carved from foam and sealed with fiberglass, was designed to replicate the bathymetry of the Devils Hole shelf; however, the AMFCF shelf is ~50 cm below the water surface, somewhat deeper than modern-day Devils Hole, consistent with historic (pre-pumping) water levels. Water in the AMFCF refuge tank is derived alternately from two dedicated production wells: Well P-1 (~73 m deep) and Well P-9 (~60 m deep), both located <10 m from the AMFCF refuge tank. The wells are cased with PVC and screened within alluvium, accessing the same regional aquifer that supplies Devils Hole. With the exception of higher temperature (~40°C) and lower bioavailable nitrogen concentrations (Table 1), the wells possess physicochemistry similar to that of Devils Hole water. Water quality parameters in the AMFCF tank (pH, dissolved oxygen, temperature, and flow rate) are continuously monitored and controlled using specialized hardware and software. During routine operation, raw well water is added to an aeration tank at a rate of 10–30 L/min, where it cools and is mixed with recirculated refuge water prior to being delivered to the refuge tank. Water in the refuge tank is continuously recirculated (150–280 L/min) within the system, during which it undergoes particulate and biological filtration, continued temperature adjustment, and UV disinfection prior to mixing with raw well water in the aeration tank. Ambient air temperature and light are controlled manually by opening or closing louvers in the AMFCF enclosure building roof. The AMFCF has the same directional orientation as Devils Hole and light data from Devils Hole are used by AMFCF managers to simulate the duration of direct light in AMFCF by adjusting the louvers seasonally, although the photoperiod at AMFCF is lengthened compared to Devils Hole due to a glass garage door on the southwest wall of the refuge tank enclosure.

**Table 1:** Major physicochemical characteristics of waters from Devils Hole (DH), Ash Meadows Fish Conservation Facility (AMFCF), and Well P-9.

	Sample				
	DH Pool	DH Shelf	AMFCF Pool	AMFCF Shelf	Well P-9
Physical Measurements					
Temp. (°C)	33.55	33.50	30.35	30.78	38.42
pH	7.22	7.33	7.71	7.77	7.58
Conductivity (μS/cm)	716	700	640	641	868
DO (mg/L) [% sat.]	2.53 [35.7%]	2.64 [37.3%]	5.02 [73.2%]	5.64 [82.9%]	3.90 [59.5%]
Salinity (ppt)	n.a.	n.a.	0.33	0.33	0.33
TDS (g/L)	n.a.	n.a.	0.400	0.400	0.449
Alkalinity: Bicarb as CaCO_3_ (mg/L)	265	n.a.	229	n.a.	238
Total Organic Carbon (mg/L)	0.141	n.a.	0.209	n.a.	0.167
Dissolved Organic Carbon (mg/L)	0.127	n.a.	0.231	n.a.	0.128
Dissolved Ions (mg/L)					
Cl	22.8	n.a.	20.1	n.a.	20.0
SO_4_	89.9	n.a.	76.6	n.a.	77.5
As	0.125	n.a.	0.0188	n.a.	0.0147
Ca	52.3	n.a.	42.7	n.a.	45.6
Mg	21.5	n.a.	18.6	n.a.	18.8
K	7.9	n.a.	9.8	n.a.	9.6
SiO_2_	23.7	n.a.	33.9	n.a.	34.1
Na	69.1	n.a.	69.5	n.a.	69.3
Dissolved Nutrients (mg/L)					
N as NO_3_	0.143	0.138	0.022	0.022	0.041
N as NO_2_	<0.002	<0.002	<0.002	<0.002	<0.002
N as NH_3_	0.004	0.004	0.004	0.004	0.003
P as O-PO_4_	0.003	0.003	0.003	0.003	0.004

n.a.–Not applicable, sample not collected

### Sample collection and water physicochemistry

Sampling at Devils Hole was conducted under US National Park Service (NPS) permit DEVA-2009-SCI-005 and at AMFCF under US Fish and Wildlife Service (USFWS) permit 84550-15-03, both issued to D.P.M. Both sites were sampled on 11-September, 2015. During the hours of sampling (11:00–13:00 UTC-8 at Devils Hole and 15:00–17:00 UTC-8 at AMFCF), Devils Hole received only indirect sunlight, whereas AMFCF received direct sunlight. Water physical parameters (temperature, pH, conductivity, total dissolved solids [TDS], salinity, and dissolved oxygen [DO]) were measured via model 6920-V2 sondes (Yellow Springs Instruments, Yellow Springs, OH), deployed in the water overlying the shallow shelf and deep pool of Devils Hole and AMFCF or via flow cell for Well P-9, the well in use at the time of sample collection. Meteoric conditions during sampling were typical of the season at this location, characterized by calm winds, clear skies, and an air temperature of 30°C to 40°C. Water samples were collected via peristaltic pumps (GeoPump II, Geotech, Denver, CO) and autoclaved, platinum-cured silicone tubing (Masterflex LS-14) flushed with 2 L of sample prior to collection. Shelf water samples were collocated with sonde deployments at ~20 cm below water surface; whereas, pool samples (also ~20 cm below surface) were not. Samples from Well P-9 were collected by attaching sterile tubing directly to a dedicated sampling port at the wellhead. At each site, unfiltered water was collected for aqueous chemical analysis (total organic carbon [TOC], alkalinity measurements) and microbial cell counts (Petroff-Hausser counting chamber). Filtrate from 0.22 μm Sterivex polyethersulfone filters (EMD Millipore, Darmstadt, Germany) was collected for dissolved aqueous chemistry analysis (dissolved organic carbon [DOC], cations, anions, nutrients, metals, and trace elements) after passage of 1 L to flush the sampling lines. Microbial biomass from ~8–10 L of water (Devils Hole pool and shelf, AMFCF pool and shelf, and Well P-9) was concentrated onto 0.22 μm Sterivex polyethersulfone filters and eight surface sediment slurries, accounting for the top ~2 cm of sediment, were collected from each of the shelves using a plastic turkey baster (provided by the National Park Service) for prokaryotic community analysis. These samples were checked for the presence of fish embryos, of which none were found, transferred to sterile 50 mL polypropylene conical centrifuge tubes, and placed on dry ice.

Samples for nitrogen and phosphorus analysis were preserved by addition of 2 mL of 25% H_2_SO_4_. TOC and DOC samples were collected in acid-washed, combusted amber glass vials and preserved with 6M HCl to a pH of ~2. Aqueous chemistry samples were stored on ice after collection and during transport. Water samples were shipped to the Desert Research Institute Water Analysis Laboratory (Reno, NV) for dissolved ammonia, nitrate, nitrite, and phosphate analysis (Methods SM 4500-NH3-H, SM 4500-NO3-F, and SM 4500-PE [32]); to Anatek Labs (Moscow, ID) for TOC and DOC analysis (Method SM 5310-B [32]); and to ACZ Laboratories (Steamboat Springs, CO) for alkalinity and dissolved ion analysis (Methods SM 2320-B, SM 4500Cl-E [32], EPA 200.7, EPA 200.8, and EPA 300.0).

### DNA extraction, library preparation, and 16S rRNA gene sequencing

Total genomic DNA was isolated from one 0.22 μm Sterivex filter per location and sediment slurries (300 μL) using MoBio PowerSoil DNA Isolation Kits (MoBio, Carlsbad, CA) following manufacturer’s instructions, with the addition of a freeze-thaw step prior to bead-beating (30 min at -80°C, 10 min at 65°C). Library preparation and Illumina sequencing were performed at the WestCore DNA Core Facility (Black Hills State University, Spearfish, SD). DNA was quantified using a Qubit 2.0 Fluorometer (Life Technologies, Carlsbad, CA), and up to 15 ng was used to prepare sequencing libraries according to the Illumina 16S Metagenomic Sequencing Protocol 15044223 Revision B (Illumina, San Diego, CA). Library preparation was carried out via PCR using modified primer sequences targeting the V4 hypervariable region of the 16S rRNA gene found in Prokaryotes (F515 [5’-GTGYCAGCMGCCGCGGTAA-3’] and 806R [5’-GGACTACHVGGGTWTCTAAT-3’] [33]). Amplified libraries were barcoded using the Illumina Nextera XT Index Kit. Final libraries were quantified, normalized, and pooled. The final library pool was then size-selected and gel-purified (2% low-melting-point agarose), and sequenced in one Illumina MiSeq instrument run using the 2x250 MiSeq Reagent Kit v2. The raw 16S rRNA gene sequences were deposited in the European Nucleotide Archive under project accession number PRJEB24636.

### Prokaryotic community analysis

Sequencing reads were demultiplexed on Illumina BaseSpace using MSR V2.4 software. The remaining analyses were conducted in QIIME 1.9.1 [34]. In total, 1,361,167 paired-end reads were generated (S1 Table). Paired-end reads were merged according to the fastq-join method [35] using default parameters. Merged reads containing ambiguous (‘N’ characters) and low-quality base calls (Phred score <30) were removed. Chimeric sequences were identified with the usearch61 algorithm [36] and removed. Operational taxonomic units (OTUs) were generated from the 1,006,989 high-quality nonchimeric sequences, based on 97% sequence similarity, and taxonomy assignments made with a subsampled open-reference OTU-picking strategy using usearch61 and uclust [36] against the Greengenes 13_8 database [37, 38]. OTUs supported by less than 0.005% of all sequences (50 sequences per OTU) were removed. A phylogenetic neighbor-joining tree [39], based on PyNAST-aligned OTU sequences [40], was generated and used for alpha and beta diversity metrics. Lastly, the OTU table was rarefied to a depth of 10,000 sequences per sample to account for differences in sequencing depth. Alpha diversity metrics (observed OTU richness, Chao1 estimated richness, Faith’s Phylogenetic Diversity index, and Shannon’s index) and pairwise Bray-Curtis and UniFrac [41] distances between samples were calculated from 100 rarefied OTU tables.

### Statistical analyses

Statistical analyses were conducted with R [42]. A two-tailed Student’s *t*-test was conducted to identify significant differences in planktonic cell concentrations between Devils Hole, AMFCF, and Well P-9. Principal component analysis and analysis of similarity tests (ANOSIM) of weighted and unweighted UniFrac distances and Bray-Curtis distances were conducted using vegan [43]. Clustering of prokaryotic communities was evaluated by constructing a dendrogram based on unweighted pair group method with arithmetic mean (UPGMA)-clustering of weighted and unweighted UniFrac distances. Node support values were calculated from 100 rarefied OTU tables of 10,000 sequences per sample. Similarity percentage (SIMPER) analysis, based on Bray-Curtis dissimilarity, was performed to identify OTUs responsible for differences between groups of samples.

## Results

### Environmental chemistry

Physicochemical conditions, summarized in Table 1 and S2 Table, were measured on both the shelf and pool at each site and were internally consistent between locations on the day of sampling. Water temperatures were higher in Devils Hole (33.55 and 33.50°C, pool vs. shelf) than in AMFCF (30.35 and 30.78°C, pool vs. shelf) (Table 1 and S1 Table), and higher still in Well P-9 (38.4°C). pH measurements averaged 7.28 in Devils Hole, 7.74 in AMFCF, and 7.58 in Well P-9. Conductivity was relatively low and consistent across all five sampling sites (640–868 μS cm^-1^). Average dissolved oxygen concentrations were 2.6 mg L^-1^ in Devils Hole (36.5% saturation), 5.3 mg L^-1^ in AMFCF (78.1% saturation), and 3.9 mg L^-1^ (59.5% saturation) in Well P-9 at the time of sampling. Consistent with a shared carbonate-buffered aquifer source for Devils Hole and AMFCF, major ions and alkalinity values were equivalent to within a few percent across the dataset. Alkalinity values ranged from 229 mg L^-1^ in AMFCF to 265 mg L^-1^ in Devils Hole, and all water samples where characterized by sulfate > sodium > calcium > magnesium/chloride. Dissolved organic carbon concentrations were low (0.127, 0.128, and 0.231 mg L^-1^ in Devils Hole, Well P-9, and AMFCF, respectively). Bioavailable nitrogen concentrations (primarily dissolved nitrate) were very low in AMFCF and Well P-9 (0.022 and 0.041 mg L^-1^) compared to Devils Hole; which, while also low, were greater by nearly an order of magnitude (0.143 and 0.138 mg L^-1^, pool and shelf). Nitrite (<0.002 mg L^-1^), ammonia (0.003–0.004 mg L^-1^), and phosphorus (0.003–0.004 mg L^-1^) concentrations were very low, approaching detection limits, in all samples. Correspondingly, N:P molar ratios for the Devils Hole shelf, Devils Hole pool, AMFCF shelf, AMFCF pool, and Well P-9 were 105:1, 108:1, 19:1, 19:1, and 24:1, respectively. Devils Hole, AMFCF, and Well P-9 had remarkably similar dissolved metal concentrations overall (Table 1 and S1 Table). However, dissolved arsenic in Devils Hole (0.125 mg L^-1^) was nearly an order of magnitude higher than all AMFCF-associated sites. Conversely, the concentration of silica in Devils Hole (23.7 mg L^-1^) was lower than in AMFCF and Well P-9 (33.9 and 34.1 mg L^-1^).

### Prokaryotic diversity and community structure

Planktonic microbial density was low in all samples from Devils Hole and AMFCF, averaging 7.9E+4 to 9.2E+4 cells mL^-1^, with no pattern between sites, and was significantly higher (1.79E+5 cells mL^-1^, p≤0.0012, Student’s *t*-test) in Well P-9 (S3 Table). In total, 2,039 operational taxonomic units (OTUs) were identified at 97% sequence similarity from the 862,307 quality-filtered sequences generated from the twenty-one samples that comprised this study (S1 Appendix). The two most abundant OTUs, an unclassified cyanobacterium (OTU_500, affiliated with the genus *Oscillatoria*) and an unclassified bacterium in the Verrucomicrobiaceae family of Verrucomicrobia (OTU_1850), accounted for 4.8% and 4.7% of all sequences. The Cyanobacteria OTU was most abundant in Devils Hole water and sediment samples (13.2% mean relative abundance), and nearly completely absent (<0.01%) from AMFCF and Well P-9 samples. Conversely, the Verrucomicrobiaceae OTU was found in high abundances in AMFCF water samples (31.7% mean relative abundance), but was present only at low abundances (≤2.4%) in all Devils Hole samples and AMFCF sediments, and was completely absent from Well P-9.

**Fig 2:**
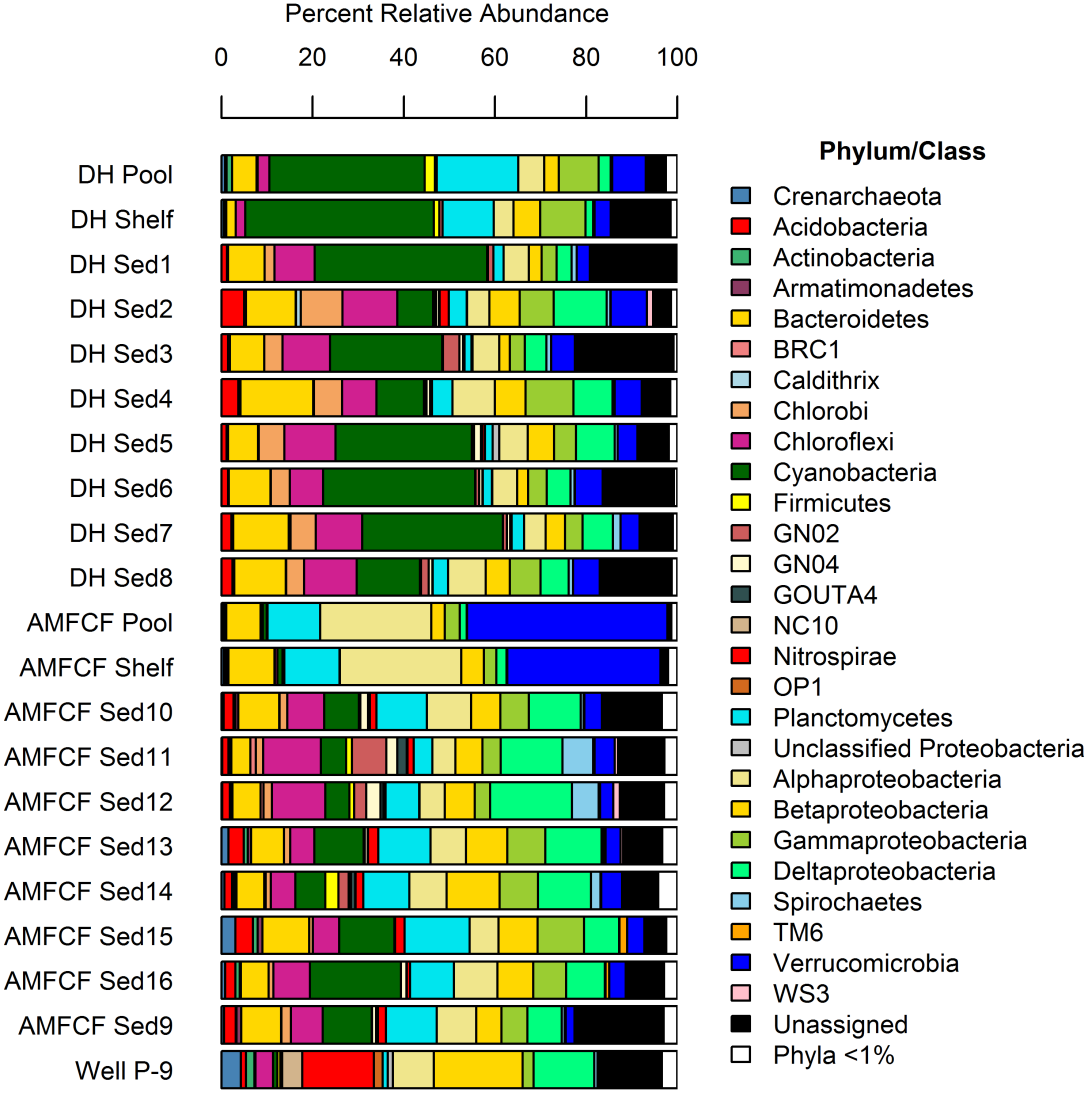
Prokaryotic community composition. Phylum-level taxonomic bar chart for prokaryotic communities from Devils Hole (DH), Ash Meadows Fish Conservation Facility (AMFCF), and Well P-9 constructed from the final unrarefied OTU table (S1 Appendix). Planktonic samples (“pool”, “shelf”, and “Well P-9”) and sediment samples (“Sed1-Sed16”), each representing a discrete sample, are noted. The Proteobacteria were subdivided into classes. Only microbial groups with abundances ≥ 1% are displayed. Groups with < 1% abundances are included in aggregate as “Phyla < 1%”.

Devils Hole and AMFCF host diverse prokaryotic communities (Fig 2), with 44 bacterial and archaeal phyla detected, including 23 candidate divisions. Devils Hole planktonic samples were dominated by Cyanobacteria (37.7% mean relative abundance), with Plantomycetes (14.5%), Gammaproteobacteria (9.3%), unassigned taxa (8.7%), Verrucomicrobia (5.5%), and Alphaproteobacteria (5.0%) accounting for large proportions of community structure. Devils Hole sediment communities were dominated by Cyanobacteria (23.6%), unassigned taxa (11.9%), Bacteroidetes (10.2%), Chloroflexi (9.9%), Deltaproteobacteria (6.8%), Alphaproteobacteria (6.3%), Gammaproteobacteria (5.5%), Verrucomicrobia (5.3%), and Chlorobi (5.1%). AMFCF planktonic communities were dominated by Verrucomicrobia (38.7%), Alphaproteobacteria (25.5%), Planctomycetes (11.8%), and Bacteroidetes (8.9%). In contrast, AMFCF sediments were dominated by Deltaproteobacteria (11.3%), unassigned taxa (10.1%), Planctomycetes (9.9%), Cyanobacteria (9.8%), Chloroflexi (8.0%), Betaproteobacteria (7.7%), Alphaproteobacteria (7.6%), Bacteroidetes (7.2%), and Gammaproteobacteria (6.7%). Well P-9 was dominated by Betaproteobacteria (19.5%), Nitrospirae (15.7%), unassigned taxa (13.9%), Deltaproteobacteria (13.3%), and Alphaproteobacteria (9.0%). Across the entire dataset, Bacteria far outnumbered Archaea, but this domain was present and accounted for <0.01–3.16% relative abundance in Devils Hole and AMFCF prokaryotic communities. Twenty-one phyla were present at <1% relative abundance across all sites.

Alpha diversity metrics (observed OTU richness, Chao1 estimated richness, Faith’s Phylogenetic Diversity [Faith’s PD], and Shannon’s index) were calculated from 100 rarefactions of 10,000 sequences per sample (S4 Table). The Devils Hole pool sample had significantly higher alpha diversity values than all other planktonic samples (Devils Hole shelf, AMFCF pool and shelf, and Well P-9) (p<0.0001 for all comparisons, Student’s *t*-test). The AMFCF shelf sample was significantly more diverse than the AMFCF pool sample and the Well P-9 sample (p<0.0001). Sediment samples, as site-specific groups, were more diverse than their planktonic counterparts (p<0.0001). AMFCF sediments collectively were more diverse than DH sediments (p<0.0001). The least diverse sample in the dataset was the Devils Hole shelf sample, with an observed OTU richness of 101.5 ± 0.59 OTUs.

**Fig 3:**
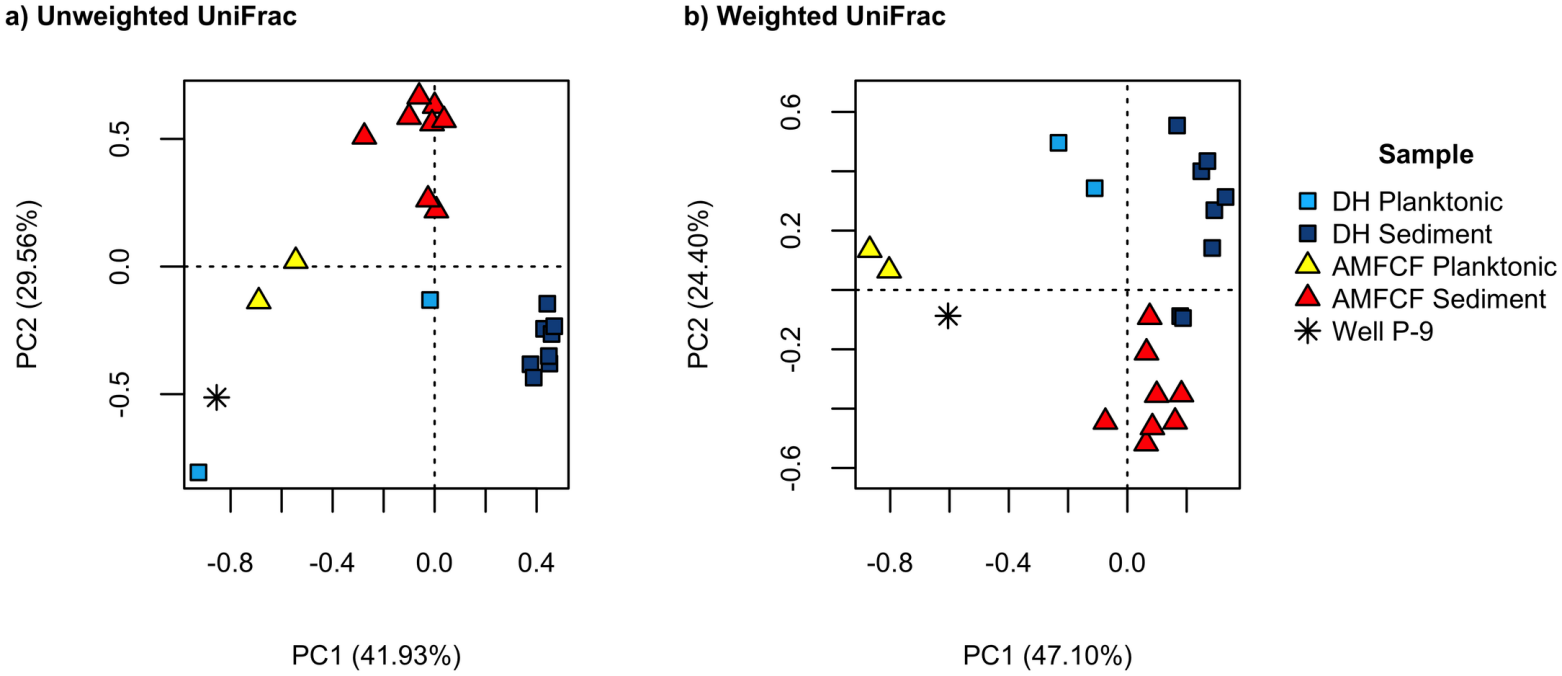
Principal component analysis. Principal component analysis ordinations of a) pairwise abundance-unweighted UniFrac distances and b) pairwise abundance-weighted UniFrac distances for all samples show separation of samples by sample location and sample type (planktonic vs. sediment). Individual samples are colored according to sample type (planktonic vs. sediment: Devils Hole (DH) planktonic and sediment samples are shown as light blue and dark blue squares, respectively; Ash Meadows Fish Conservation Facility (AMFCF) planktonic and sediment samples are shown as yellow and red triangles, respectively; and the Well P-9 sample is shown as an asterisk.

To evaluate the similarity between prokaryotic communities, particularly between Devils Hole and AMFCF, pairwise unweighted UniFrac, weighted UniFrac, and Bray-Curtis distances were calculated, UPGMA clustering was performed, and ordination diagrams were generated. Principal component analysis of abundance-unweighted (Fig 3a) and abundance-weighted (Fig 3b) UniFrac distances show the distribution of prokaryotic communities in the statistical space formed by the first two components. Unweighted and weighted analyses showed separation of communities based upon both sample location and substrate type (planktonic vs. sediment). Abundance-unweighted and –weighted UPGMA clustering dendrograms (S1 and S2 Figs) validated these observations, with each cluster of samples supported by 100% jackknife support, although the DH Shelf and DH Pool samples clustered independently in the abundance-unweighted dendrogram. The Well P-9 sample clustered independently of all other samples in both of the ordinations (Fig 3a and 3b) and dendrograms. In the abundance-weighted ordination and dendrogram (Fig 3b and S2 Fig), Devils Hole planktonic samples formed their own distinct cluster and were more similar to all sediment samples than to AMFCF planktonic samples. Sediment samples clustered in a site-specific manner.

Analysis of similarity (ANOSIM) tests were conducted to identify significant differences in taxonomic similarity (Bray-Curtis distances), qualitative phylogenetic similarity (abundance-unweighted UniFrac distances), and quantitative phylogenetic similarity (abundance-weighted UniFrac distances) between planktonic and sediment samples from Devils Hole and AMFCF (S5 Table). Taxonomic and phylogenetic similarity differed significantly between sample sites (p<0.01). Sediment samples were taxonomically and phylogenetically significantly different between Devils Hole and AMFCF (p<0.05), whereas the planktonic samples between the two sites were not significantly different.

**Fig 4:**
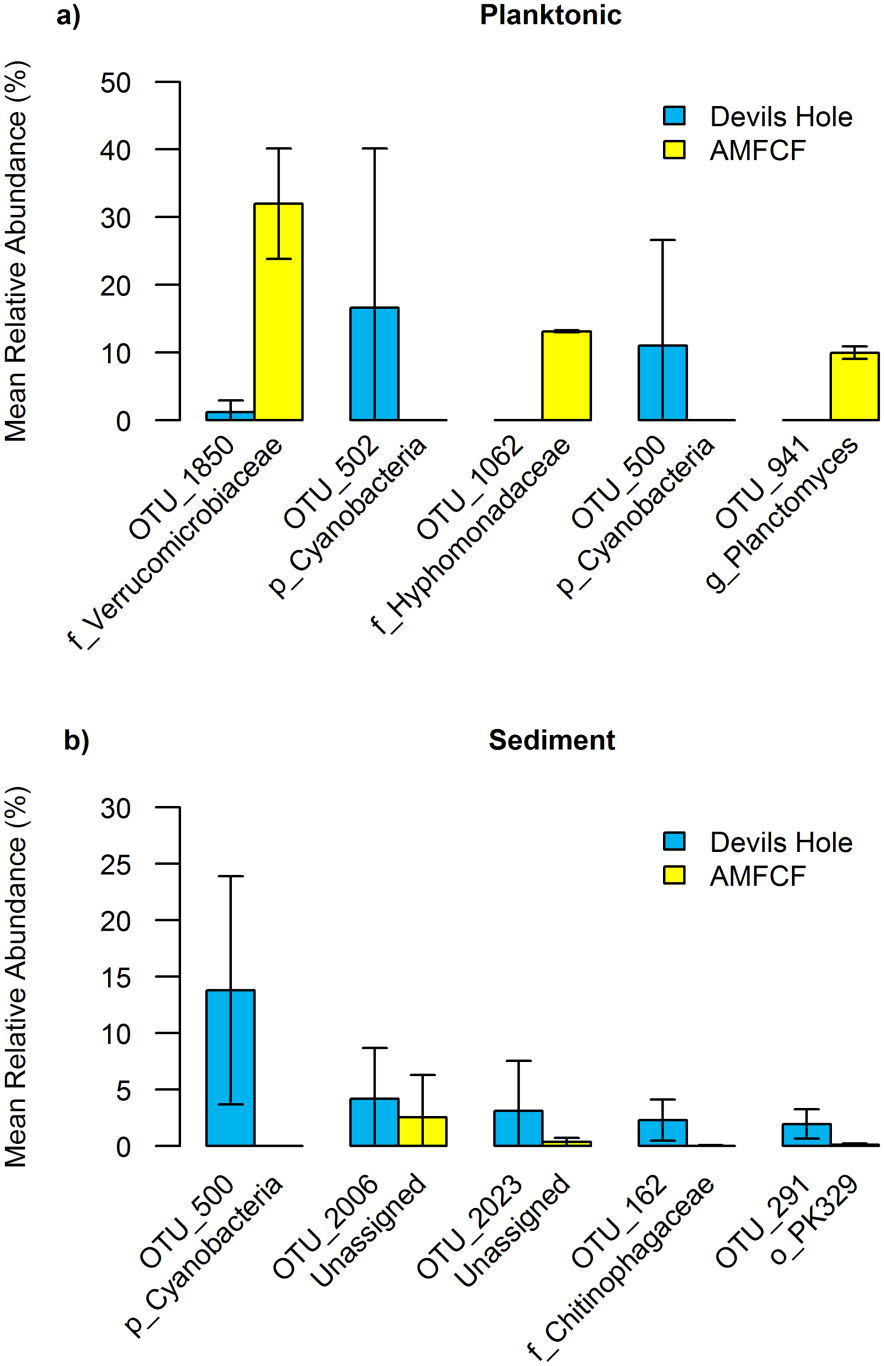
Relative abundances (mean and SD) of the top 5 differentially abundant operational taxonomic units identified by SIMPER analysis. Differentially abundant OTUs between **a)** planktonic samples and **b)** sediment samples between Devils Hole and Ash Meadows Fish Conservation Facility (AMFCF), along with OTU ID and taxonomy, are shown. Prefixes (p_, o_, f_ and g_) denote phylum, order, family, and genus level OTU identities. See S6 Table for the percent contribution of each OTU to the dissimilarity between each group of samples.

SIMPER analysis was performed to identify the top five OTUs that contributed to the dissimilarity between Devils Hole and AMFCF sediment and planktonic samples (Fig 4, S6 Table). Prokaryotic community dissimilarities (42.82% cumulative contribution) between Devils Hole and AMFCF planktonic samples were driven by two unclassified Cyanobacteria OTUs (OTU_502 and OTU_500), which were abundant in Devils Hole planktonic samples (16.64% and 11.03% mean relative abundance) and undetected in AMFCF planktonic samples, and an OTU in the Verrucomicrobiaceae family (OTU_1850), an OTU in the Hyphomonadaceae family (OTU_1062), and an OTU in the *Planctomyces* genus (OTU_941), which were more abundant in AMFCF planktonic samples (31.97%, 13.15%, and 9.99%, respectively) compared to Devils Hole planktonic samples (1.21%, 0.01%, and undetected, respectively). Two phyla were detected in Devils Hole planktonic samples but absent from AMFCF planktonic samples (Fusobacteria and Gemmatimonadetes, both <1%). Conversely, six phyla were detected in low abundances in AMFCF planktonic samples (Elusimicrobia, GOUTA4, OC31, OP11, OP3, and SBR1093, all <1%) but were absent in Devils Hole planktonic samples.

Dissimilarities between Devils Hole and AMFCF sediment samples (15.16% cumulative contribution) were attributed to an unclassified Cyanobacteria OTU (OTU_500), two unassigned OTUs (OTU_2006 and OTU_2023), an OTU in the Chitinophagaceae family (OTU_162), and an OTU in the PK329 order of Chlorobi (OTU_291), which were found in higher abundance in Devils Hole sediments (13.81%, 4.21%, 3.11%, 2.29%, and 1.95%, respectively) compared to AMFCF sediments (0.0038%, 2.54%, 0.36%, 0.033%, and 0.12%, respectively). Three phyla were detected in Devils Hole sediments at low abundances (Euryarchaeota, Caldithrix, and FCPU426, all <0.5%) but were absent from AMFCF sediments. Conversely, the phylum OP11 was detected in a single AMFCF sediment sample but no other sediment samples from either AMFCF or Devils Hole (<0.01%).

## Discussion

Organisms which depend upon isolated aquatic systems of closed basins represent a unique ecosystem management challenge; whereby an entire species, being confined to a single location, is by definition acutely susceptible to extirpation by manmade or natural catastrophes [6]. Examples of species loss among desert fishes, including in and around the spring system that is the focus of this work (Ash Meadows National Wildlife Refuge), abound [6, 44]. In a number of cases, the establishment of refuge populations of desert fishes has successfully forestalled the otherwise inevitable demise of unique species [45]. Of all endangered desert fishes, the Devils Hole pupfish is probably the best known. Previous attempts to establish refuge populations of the Devils Hole pupfish have failed for various reasons [18, 19]; but in 2013, a much more sophisticated example, the Ash Meadows Fish Conservation Facility, was completed. In this study, to gain insights into the degree to which the AMFCF simulates the Devils Hole ecosystem and to establish a baseline dataset against which to calibrate future measurements, we evaluate the limnological status of Devils Hole and AMFCF in parallel with a comprehensive examination of planktonic and benthic prokaryotic microbiology.

### Water physicochemistry

While physical parameters in Devils Hole were consistent with those previously reported for the site [16], water temperature and the concentrations of dissolved oxygen, silica, and arsenic differed in the AMFCF when compared to those of Devils Hole. Water temperature, for example, was ~3°C lower at AMFCF than at Devils Hole and dissolved oxygen was roughly double (Table 1). It should be noted, however, that interpretations from these data must be tempered by the fact that measurements represent a snapshot in time applied to dynamic environments. Most significantly, at their respective times of sample collection (11:00–13:00 UTC-8 at Devils Hole and 15:00–17:00 UTC-8 at AMFCF), Devils Hole was only indirectly lit whereas AMFCF was receiving direct sunlight. In both Devils Hole and AMFCF, dissolved oxygen concentrations over the shallow shelves fluctuated in response to light-driven diurnal effects and ranged from 2.54 mg L^-1^ during the majority of the day to 6.95 mg L^-1^ at 15:30 UTC-8 (35.8–98.4% saturation) in Devils Hole Shelf (S1 Appendix, Sheet 4), and from 4.83 mg L^-1^ to 6.46 mg L^-1^ at 17:30 UTC-8 (70.4–94.6% saturation) in AMFCF Shelf (S1 Appendix, Sheet 6). Dissolved oxygen concentrations did not fluctuate diurnally in the Devils Hole and AMFCF pools (S1 Appendix, Sheet 5 and 7). Although differences in temperature and dissolved oxygen are instituted intentionally to optimize Devils Hole pupfish recruitment and survivorship, they are known to result in morphological changes to the fish (e.g. the development of pelvic fins [46]) and could potentially lead to unanticipated genetic and epigenetic effects over time. Arsenic concentrations were in exceedance of the 0.01 mg L^-1^ drinking water maximum contaminant level in all samples. The much higher concentration of this element in Devils Hole water (0.125 mg L^-1^) compared to that of AMFCF (0.0188 mg L^-1^) and Well P-9 (0.0147 mg L^-1^) is notable as arsenic is known to support bacterial energy requirements through dissimilatory reduction and oxidation [47], and has been shown to fuel anoxygenic photosynthesis in *Oscillatoria*-like cyanobacteria [48]. Collectively or individually, in addition to their potential influences on fish, such physicochemical differences could alter prokaryotic community structure and function, thereby influencing environmental chemistry and nutrient dynamics of the ecosystem.

Measured differences in dissolved nutrient and carbon content suggest that productivity of the two sites is controlled differently. Although concentrations of nutrients were low in waters from both sites, AMFCF was substantially lower in bioavailable nitrogen (primarily nitrate, 0.022 vs. 0.141 mg L^-1^) and enriched in dissolved organic carbon relative to Devils Hole (0.231 vs. 0.127 mg L^-1^). Nitrate concentrations at Devils Hole (Table 1, 0.143 and 0.138 mg L^-1^, pool and shelf) were consistent with historic data from the US Geological Survey 1984–1990 (n = 25, $\bar{\text{x}}=0.14$ mg L^-1^, SD 0.0176, [49]), when the numbers of Devils Hole pupfish were much higher (annual maxima ranging from ~400–575 individuals [1] vs. 131 in the fall of 2015). N:P molar ratios in Devils Hole water were 105:1 and 108:1 (shelf and pool), and 19:1 for AMFCF (shelf and pool). Klausmeier and colleagues [50] have shown that optimal intracellular N:P ratios in phytoplankton are dictated by environmental conditions and nutrient availability; however, N:P ratios in excess of 50:1 often indicate phosphorus limitation [51], expanding on the ‘optimal’ 106:16:1 C:N:P ratio proposed for oceanic phytoplankton [52]. Consistent with these more recent interpretations, at the time of sampling, Devils Hole clearly exhibited phosphorus limitation, as has been previously reported [10, 53]. In marked contrast, AMFCF exhibited N:P ratios indicative of neither nitrogen nor phosphorus limitation. Rather, co-limitation likely results from the very low concentrations of both.

As the AMFCF has much lower bioavailable nitrogen concentrations than Devils Hole (today and in decades past), further consideration of the source of nutrient inputs into these ecosystems is warranted. It is known, for example, that allochthonous nutrient and fixed carbon contributions from dry deposition, fecal pellets, and meteoric runoff are considerable at Devils Hole, contributing an estimated 60% of the total energy available to the food web [16]. Conversely, all of these contributions are minimized at AMFCF due to its disconnection from surface drainage and artificial enclosure. Allochthonous nutrient contributions are thus potentially an overlooked component contributing to the inability of AMFCF to replicate trophic control and biogeochemistry of Devils Hole. The striking divergence in limiting nutrient likely leads to corresponding differences in trophic structure and control between the two sites; in all probability, both influenced by and reflected in corresponding differences in prokaryotic community structure.

### Prokaryotic community differences

Devils Hole and AMFCF are microbiologically diverse, with over 40 prokaryotic phyla represented (Fig 2), more than half of which belong to candidate divisions (phylum-level lineages of Bacteria and Archaea with no cultivated representatives), which accounted for 1.9–26.7% (including unassigned OTUs) of prokaryotic community structure (S1 Appendix Sheets 1 and 3). Although the majority of taxa detected belonged to the domain Bacteria, Archaea accounted for <0.01–3.16% of all prokaryotic communities, of which almost all were representatives of the Cenarchaeales order of Thaumarchaeota, which have been previously reported as dominant archaeal community members from nearby springs of the Ash Meadows National Wildlife Refuge [27]. This remarkable prokaryotic biodiversity may exceed that from the Cuatro Cienegas Basin, an oligotrophic spring system in the Chihuahuan Desert; where 10 bacterial phyla were detected in 16S rRNA gene clone libraries, a result that was used to support the authors’ characterization of the site as an "endangered oasis of aquatic microbial biodiversity” [54].

In this dataset, despite similarities in the number and identity of the phyla represented, planktonic prokaryotic communities of AMFCF were dominated by different microbial taxa than Devils Hole. Unweighted UniFrac analysis, a statistical consideration of presence/absence of organisms, indicated that prokaryotic communities in Devils Hole and AMFCF were distinct and composed of different taxa (Fig 3). Weighted UniFrac analysis, which further incorporates the abundance of organisms, revealed similar patterns, indicating that a subset of community members in each ecosystem were differentially abundant among the two sites. Although not significantly different (S5 Table, likely due to a low sample number), planktonic prokaryotic communities from Devils Hole and AMFCF samples were composed of distinct taxa. Conversely, ANOSIM results indicated that sediment communities from Devils Hole and AMFCF were significantly different. Devils Hole planktonic prokaryotic communities were dominated by two unclassified OTUs of Cyanobacteria (OTU_502 and OTU_500), each of which had 99% sequence identity to 16S rRNA gene sequences previously detected from Devils Hole (GenBank Accession Number KC358607) and 97% sequence similarity to *Oscillatoria duplisecta* type strain ETS-06 (GenBank Accession Number AM398647) isolated from Euganean thermal spring muds in Italy [55]. These abundant taxa (16.64% and 11.03% mean relative abundance) were virtually absent in AMFCF planktonic and sediment samples (Fig 4), even though Cyanobacteria comprised 5–20% of AMFCF sediment communities. In Devils Hole sediments, cyanobacterial OTU_500 was abundant (15.16%) but OTU_502 was virtually absent. Cyanobacteria, a primary food source in summer for the Devils Hole pupfish, are responsible for much of the primary production in Devils Hole and contribute an estimated 15.8 J mg^-1^ of energy to the food web [15, 16]. *Oscillatoria*, a genus of filamentous Cyanobacteria capable of fixing nitrogen autotrophically and photoheterotrophically without producing heterocysts [56, 57], have been previously reported in high abundances at Devils Hole [10, 53]. In contrast to reports of robust seasonal cyanobacterial mats from all three of the historic refuges, often dominated by *Oscillatoria spp*. (12), the very low abundance (~1%) of Cyanobacteria in our summer AMFCF planktonic samples is perplexing. However, since the AMFCF refuge tank receives 10–30 L/min of raw water from Well P-9, of which Cyanobacteria only account for 0.94% of the community, and since water in the AMFCF refuge tank is constantly recirculated, filtered, and UV disinfected, it is possible that Cyanobacteria are unable to become established members of the AMFCF planktonic community. It is also conceivable that populations of photosynthetic primary producers in AMFCF were dominated by algae and diatoms; which being eukaryotes, would have likely been missed in our 16S rRNA gene surveys. Possibly consistent with this scenario, silica was higher in AMFCF than in Devils Hole (33.9 mg L^-1^ vs. 23.7 mg L^-1^). However, the similarity in silica concentrations between AMFCF and its water supply well (Well P-9, 33.9 vs. 34.1 mg L^-1^) supports little if any loss resulting from diatom growth.

During blooms, cyanobacteria will incorporate fewer nitrogen atoms per phosphorus atom, reducing their N:P ratios [58]. If this were the case, one might expect that cyanobacteria would be abundant in AMFCF; however, the concentration of nitrate and phosphorus may be too low to allow for cyanobacterial establishment and proliferation. Although ecological controls over nitrogen fixation remain poorly understood, low bioavailable phosphorus and trace metal concentrations have been implicated as controls over nitrogen fixation in oligotrophic environments [59]. In oligotrophic Patagonian lakes, nitrogen fixation was essentially nonexistent when dissolved phosphorus concentrations fell below 8 μg L^-1^ [60]. In the eastern tropical North Atlantic, nutrient addition bioassay experiments showed that nitrogen fixation was stimulated upon addition of allochthonous material (dust), which provided a source of phosphorus and iron [61]. Since dissolved phosphorus concentrations were 3 μg L^-1^ in all of the samples from this study, this could explain the lack of nitrogen-fixing cyanobacterial populations in AMFCF despite its lower bioavailable nitrogen concentration. Despite low nutrient concentrations in Devils Hole, cyanobacterial standing stocks (and hence nitrogen fixation capacity) are likely to be periodically stimulated by allochthonous inputs of fixed carbon and nutrients during meteoric events [16]. Conversely, allochthonous inputs are consistently much lower at AMFCF owing to the lack of opportunity for run-off to reach the refuge pool; a situation which may serve to discourage the proliferation of cyanobacterial populations and inadvertently select for the persistence of organisms adapted to oligotrophic conditions.

Planktonic prokaryotic communities from AMFCF were dominated by an OTU in the Verrucomicrobiaceae family of Verrucomicrobia (OTU_1850), an OTU in the Hyphomonadaceae family of Alphaproteobacteria (OTU_1062), and an OTU in the *Planctomyces* genus of Planctomycetes (OTU_941). These OTUs were comparatively rare in Devils Hole and AMFCF sediments (<0.1%) and Devils Hole planktonic samples (<2%) (Fig 4). The Verrucomicrobia, common inhabitants of both eutrophic and oligotrophic terrestrial and marine environments, are metabolically flexible and have the highest genomic frequency of polysaccharide hydrolases of any bacterial phylum [62–64], which may confer a selective advantage in oligotrophic environments. The Hyphomonadaceae contain chemoorganotrophic organisms, some of which are also denitrifiers [65]. Becerra-Castro et al. [66] have shown that Proteobacteria increased in relative abundance following UV disinfection of secondarily treated wastewater, which indicates that these organisms may be particularly resistant to UV disinfection or respond favorably to chemical changes to refractory organic carbon. The persistence of Proteobacteria following UV exposure may explain the high proportion of Alphaproteobacteria in AMFCF planktonic samples compared to Devils Hole and Well P-9 planktonic samples. The *Planctomyces*, which lack peptidoglycan and reproduce by budding [67], are common inhabitants of eutrophic habitats and their abundance tends to co-occur with cyanobacterial or diatom blooms [67, 68]. The abundance of *Planctomyces* in AMFCF, an oligotrophic environment, may suggest linkages to recent cyanobacterial or diatom productivity. Given the low concentrations of dissolved nitrogen and phosphate in AMFCF, these environmental conditions may have selected for oligotrophically adapted organisms, such as the Verrucomicrobiaceae and Hyphomonadaceae.

The dissimilarities in prokaryotic community composition and diversity between Devils Hole and AMFCF could result from discrepancies in the natural inoculation pathways and exposure times between systems. It has been proposed that Devils Hole first opened to the atmosphere ~60,000 years before present [69, 70]. Therefore, since that time, Devils Hole has been susceptible to microbial introduction not only from deeply sourced groundwater, but also from airborne deposition and meteoric runoff. Conversely, in AMFCF, even though efforts have been made to inoculate the facility with algae, invertebrates, and plankton from Devils Hole since it’s initiation in 2013, the fact that AMFCF refuge receives 10–30 L/min of raw water from Well P-9, and that water in the AMFCF refuge tank is constantly recirculated, filtered, and UV disinfected (150–280 L/min), opportunities for microbial colonization have been more limited. This is especially true when one considers the degree to which the AMFCF tank is isolated from runoff and atmospheric exposure due to its location within a partially enclosed shed. Finally, being considerably younger than Devils Hole and only recently stocked with fish, the microbial ecosystem in the AMFCF may simply represent an earlier successional stage and, over time, a gradual shift to one more closely resembling that of Devils Hole may still occur.

### Implications for mesocosm design and management

As natural and anthropogenic threats to endangered species persist and increase, resulting in the reduction or destruction of critical habitat, captive propagation is becoming an increasingly important conservation strategy [71, 72]. While physicochemical conditions (e.g. pH, temperature, dissolved oxygen, basic chemistries) of these mesocosms/refuges are maintained to simulate the organism’s natural environment [73, 74], little attention is currently given to nutrient dynamics or lower trophic support. Here we have shown that AMFCF failed to replicate the dissolved nutrient chemistry and prokaryotic community structure of Devils Hole, despite efforts to simulate the physicochemical parameters of Devils Hole. Correspondingly, we propose that these factors may contribute to low annual Devils Hole pupfish recruitment observed at AMFCF. The importance of mimicking environmental conditions from endangered species habitat in mesocosms and artificial refuges remains incompletely understood; and in the absence of studies which explore microbiology and dissolved nutrient dynamics, critical time may be lost. Although more work is needed at Devils Hole and elsewhere to address these questions, the results of this study indicate that a predictive understanding of interactions between life at the base of the food web and nutrient chemistry would improve mesocosm design and management strategies at existing facilities for the stewardship of endangered species.

## Conclusion

To establish refuge populations of the Devils Hole pupfish, it is crucial to replicate environmental conditions that promote the growth of primary producers, such as Cyanobacteria, which in turn should promote the growth of the fish at all stages of life. We have shown that, while AMFCF does approximate the natural habitat of the Devils Hole pupfish in size and topography, significant differences exist in physicochemical variables and inoculation pathways: differences which may limit cyanobacterial growth, a prominent food source for the Devils Hole pupfish. Additionally, prokaryotic community structure differed substantially between Devils Hole and the AMFCF, with Cyanobacteria dominating in Devils Hole and a single OTU from the Verrucomicrobiaceae dominating in the AMFCF. We infer that the prevention of allochthonous nutrient input and microbial inocula from runoff and terrestrial sources at AMFCF, due to engineering controls, is a plausible explanation for the divergence in prokaryotic communities and nutrient status between AMFCF and Devils Hole. Given the consistency in bioavailable nitrogen concentrations over time in Devils Hole, and the fact that bioavailable nitrogen concentrations are much lower in the AMFCF, it seems reasonable that current nutrient chemistry of Devils Hole can justifiably serve as a guide for that of AMFCF. This result also suggests that modest nutrient amendment at the AMFCF to restore bioavailable nitrogen and nutrient stoichiometry to Devils Hole values may have merit as a management strategy. Long-term microbiological and nutrient monitoring/management of AMFCF, and consideration of allochthonous deposition of carbon and nutrients, would allow for the elucidation of environmental controls and facilitate informed stewardship of this endangered fish.

## Supporting information

S1 Fig Unweighted pair group with arithmetic mean (UPGMA)-cluster tree of pairwise unweighted UniFrac distances. Node support symbols: square = 100%, circle = 90–99%, triangle = 80–89%. DH—Devils Hole, AMFCF—Ash Meadows Fish Conservation Facility.
(TIF)

S2 Fig Unweighted pair group with arithmetic mean (UPGMA)-cluster tree of pairwise weighted UniFrac distances. Node support symbols: square = 100%, circle = 90–99%. DH—Devils Hole, AMFCF—Ash Meadows Fish Conservation Facility.
(TIF)

S1 Table Sequencing statistics for 16S rRNA gene libraries. (DOCX)

S2 Table Full physicochemical characteristics of waters from Devils Hole (DH), Ash Meadows Fish Conservation Facility (AMFCF), and Well P-9. (DOCX)

S3 Table Cell counts (cells/mL) for planktonic samples collected at Devils Hole (DH), Ash Meadows Fish Conservation Facility (AMFCF), and Well P-9. (DOCX)

S4 Table Alpha diversity metrics for Devils Hole (DH), Ash Meadows Fish Conservation Facility (AMFCF), and Well P-9. (DOCX)

S5 Table Results from analysis of similarity (ANOSIM) tests between planktonic communities in Devils Hole and AMFCF and between sediment communities in Devils Hole (DH) and AMFCF (Bray-Curtis, unweighted UniFrac, and weighted UniFrac dissimilarity calculated from an OTU table rarefied to 10,000 sequences per sample). (DOCX)

S6 Table SIMPER analysis showing the top 5 OTUs responsible for the Bray-Curtis dissimilarity between planktonic communities in Devils Hole (DH) and AMFCF and between sediment communities in DH and AMFCF. Mean abundances and contributions to dissimilarity were calculated from an OTU table rarefied to 10,000 sequences per sample.
(DOCX)

S1 Appendix **Sheet 1:** Unrarefied OTU table including OTU numbers, OTU sequences, taxonomy, and OTU counts. **Sheet 2:** Rarefied OTU table including OTU numbers, OTU sequences, taxonomy, and OTU counts (rarefaction depth: 10,000 sequences per sample). Uclust-assigned taxonomy was derived from the Greengenes 13_8 reference database at 97% sequence identity (k = kingdom, p = phylum, c = class, o = order, f = family, g = genus, s = species). Proposed taxonomies are indicated by square brackets. DH—Devils Hole, AMFCF—Ash Meadows Fish Conservation Facility. **Sheet 3:** Abundances of prokaryotic phyla detected in each sample. The Proteobacteria have been subdivided into classes. **Sheets 4–7:** Multiparameter sonde data (water temperature (°C), conductivity (mS/cm), pH, dissolved oxygen (% saturation), and dissolved oxygen (mg/L)) collected every fifteen minutes on 11-September, 2015, at Devils Hole Shelf (**Sheet 4**), Devils Hole Pool (**Sheet 5**), AMFCF Shelf (**Sheet 6**), and AMFCF Pool (**Sheet 7**).
(XLSX)
