# Supplementary figures and images for: A comparative study of prokaryotic diversity and physicochemical characteristics of Devils Hole and the Ash Meadows Fish Conservation Facility, a constructed analog
Source: PLoS One. 2018 Mar 15;13(3):e0194404. doi: 10.1371/journal.pone.0194404 (PMC5854365; doi:10.1371/journal.pone.0194404)

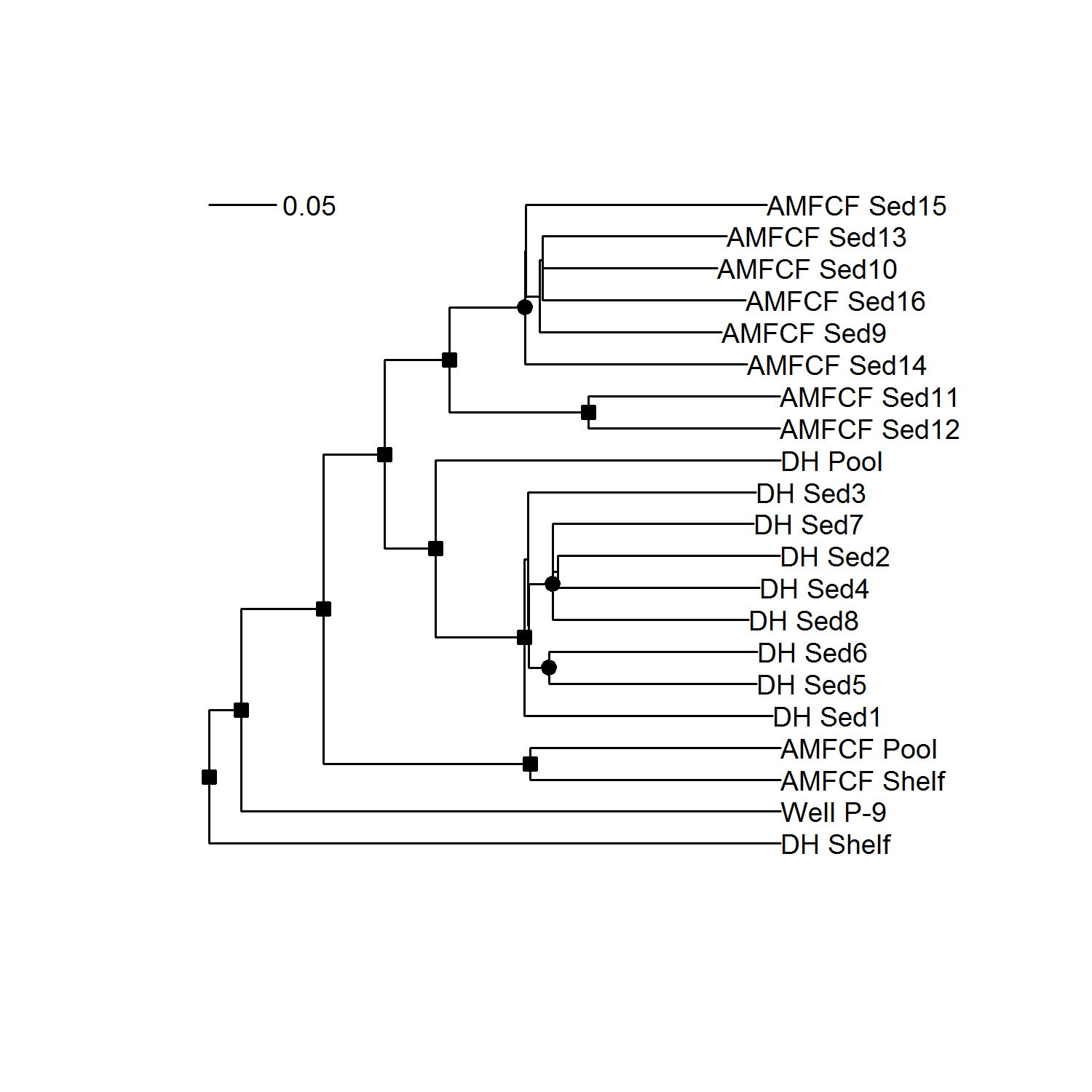

Supplement: S1 Fig — Node support symbols: square = 100%, circle = 90–99%, triangle = 80–89%. DH—Devils Hole, AMFCF—Ash Meadows Fish Conservation Facility. (TIF) [file pone.0194404.s001.tif]

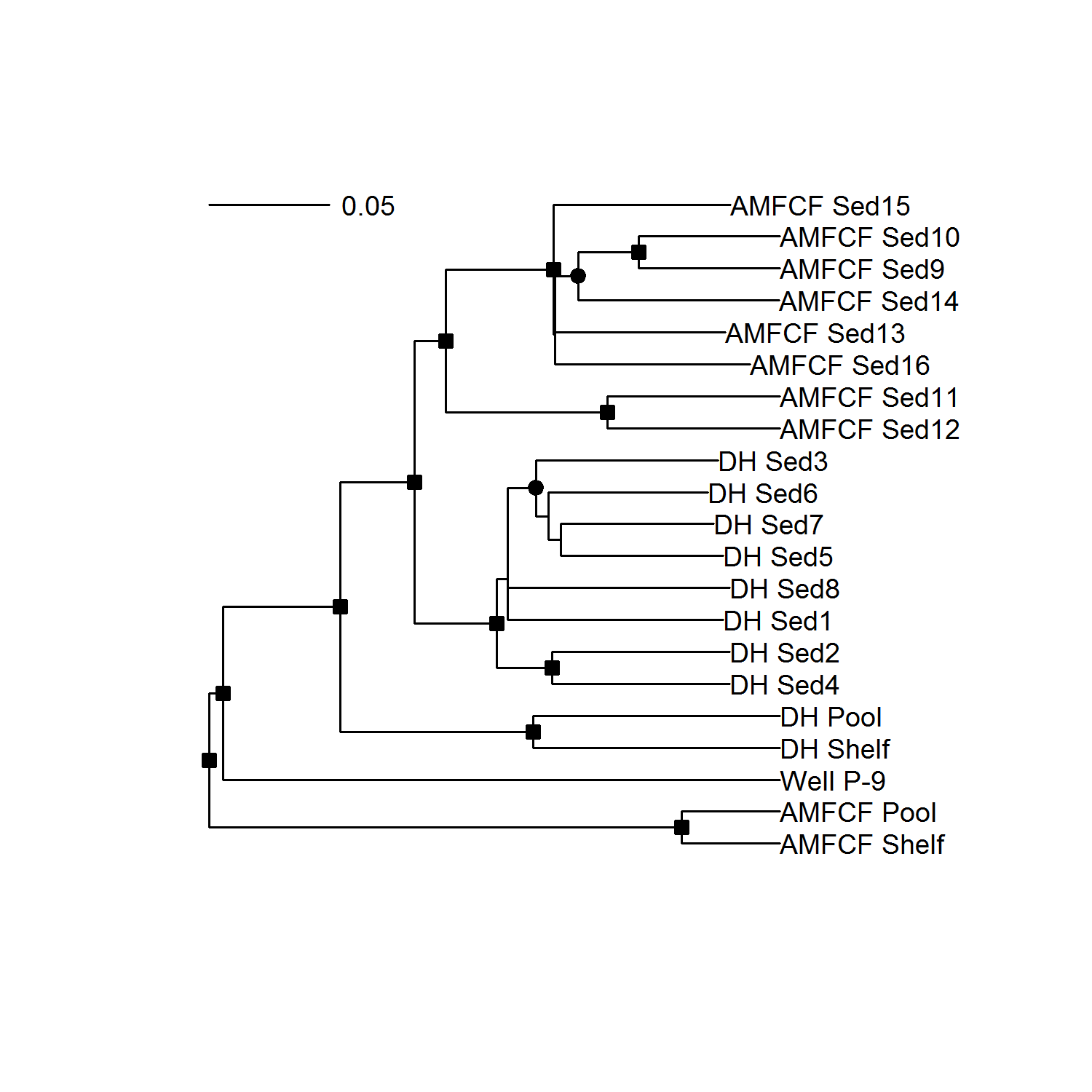

Supplement: S2 Fig — Node support symbols: square = 100%, circle = 90–99%. DH—Devils Hole, AMFCF—Ash Meadows Fish Conservation Facility. (TIF) [file pone.0194404.s002.tif]
