# Supplementary material for: A comparative study of prokaryotic diversity and physicochemical characteristics of Devils Hole and the Ash Meadows Fish Conservation Facility, a constructed analog
Source: PLoS One. 2018 Mar 15;13(3):e0194404. doi: 10.1371/journal.pone.0194404 (PMC5854365; doi:10.1371/journal.pone.0194404)
Supplement: S1 Table — (DOCX) [file pone.0194404.s003.docx]

**S1 Table. Sequencing statistics for 16S rRNA gene libraries.**

|  | ^1^Total Raw Paired-End Reads | ^2^Quality- and Chimera-Filtered Reads | ^2^Filtered Read Length (bp)  Mean (SD) | ^3^Final Read Count |
| --- | --- | --- | --- | --- |
| DH Pool | 34,038 | 21,317 | 291.8 (3.4) | 14,391 |
| DH Shelf | 66,968 | 53,114 | 291.4 (6.4) | 35,155 |
| DH Sed1 | 26,163 | 20,539 | 291.4 (6.4) | 18,935 |
| DH Sed2 | 47,442 | 32,482 | 291.5 (6.4) | 26,662 |
| DH Sed3 | 68,227 | 51,002 | 291.4 (6.5) | 46,190 |
| DH Sed4 | 91,989 | 66,891 | 291.5 (5.9) | 58,044 |
| DH Sed5 | 31,913 | 22,224 | 290.6 (9.5) | 16,996 |
| DH Sed6 | 74,796 | 54,610 | 291.2 (6.9) | 49,477 |
| DH Sed7 | 54,458 | 41,456 | 291.3 (7.2) | 37,049 |
| DH Sed8 | 105,090 | 75,018 | 291.3 (7.0) | 67,131 |
| AMFCF Pool | 93,723 | 69,524 | 291.2 (5.9) | 62,737 |
| AMFCF Shelf | 92,390 | 72,234 | 291.5 (5.3) | 63,628 |
| AMFCF Sed9 | 43,556 | 33,264 | 291.3 (7.5) | 28,819 |
| AMFCF Sed10 | 50,274 | 36,122 | 291.6 (6.1) | 31,982 |
| AMFCF Sed11 | 89,723 | 68,174 | 290.6 (9.2) | 60,462 |
| AMFCF Sed12 | 96,276 | 70,075 | 291.3 (7.6) | 61,904 |
| AMFCF Sed13 | 30,006 | 21,764 | 291.2 (7.5) | 18,854 |
| AMFCF Sed14 | 72,206 | 54,377 | 291.4 (7.5) | 48,180 |
| AMFCF Sed15 | 76,588 | 58,136 | 291.7 (6.4) | 48,763 |
| AMFCF Sed16 | 74,648 | 56,279 | 291.4 (6.7) | 49,572 |
| Well P-9 | 40,663 | 28,387 | 291.2 (7.7) | 17,376 |
| ^1^Calculated prior to paired-end joining  ^2^Calculated after paired-end joining, quality filtering, and chimera removal  ^3^Calculated after paired-end joining, quality filtering, chimera removal, and abundance-based OTU filtering | | | | |
