# Supplementary material for: A comparative study of prokaryotic diversity and physicochemical characteristics of Devils Hole and the Ash Meadows Fish Conservation Facility, a constructed analog
Source: PLoS One. 2018 Mar 15;13(3):e0194404. doi: 10.1371/journal.pone.0194404 (PMC5854365; doi:10.1371/journal.pone.0194404)
Supplement: S2 Table — (DOCX) [file pone.0194404.s004.docx]

**S2 Table. Full physicochemical characteristics of waters from Devils Hole (DH), Ash Meadows Fish Conservation Facility (AMFCF), and Well P-9.**

|  | Sample | | | | |  |  |  |  |
| --- | --- | --- | --- | --- | --- | --- | --- | --- | --- |
|  | DH Pool | DH Shelf | AMFCF Pool | AMFCF Shelf | Well P-9 |  |  |  |  |
| Physical Measurements |  |  |  |  |  |  |  |  |  |
| Temp. (°C) | 33.55 | 33.50 | 30.35 | 30.78 | 38.42 |  |  |  |  |
| pH | 7.22 | 7.33 | 7.71 | 7.77 | 7.58 |  |  |  |  |
| Conductivity (μS/cm) | 716 | 700 | 640 | 641 | 868 |  |  |  |  |
| DO (mg/L) [% sat.] | 2.53 [35.7%] | 2.64 [37.3%] | 5.02 [73.2%] | 5.64 [82.9%] | 3.90 [59.5%] |  |  |  |  |
| Salinity (ppt) | n.a. | n.a. | 0.33 | 0.33 | 0.33 |  |  |  |  |
| TDS (g/L) | n.a. | n.a. | 0.400 | 0.400 | 0.449 |  |  |  |  |
| Hardness as CaCO_3_ (mg/L) | 219 | n.a. | 183 | n.a. | 191 |  |  |  |  |
| Alkalinity: Bicarb as CaCO_3_ (mg/L) | 265 | n.a. | 229 | n.a. | 238 |  |  |  |  |
| Alkalinity: Carbonate as CaCO_3_ (mg/L) | <2 | n.a. | <2 | n.a. | <2 |  |  |  |  |
| Alkalinity: Hydroxide as CaCO_3_ (mg/L) | <2 | n.a. | <2 | n.a. | <2 |  |  |  |  |
| Total Organic Carbon (mg/L) | 0.141 | n.a. | 0.209 | n.a. | 0.167 |  |  |  |  |
| Dissolved Organic Carbon (mg/L) | 0.127 | n.a. | 0.231 | n.a. | 0.128 |  |  |  |  |
| Dissolved Ions (mg/L) |  |  |  |  |  |  |  |  |  |
| Cl | 22.8 | n.a. | 20.1 | n.a. | 20.0 |  |  |  |  |
| SO_4_ | 89.9 | n.a. | 76.6 | n.a. | 77.5 |  |  |  |  |
| Al | <0.001 | n.a. | 0.005 | n.a. | <0.001 |  |  |  |  |
| Sb | <0.0004 | n.a. | <0.0004 | n.a. | <0.0004 |  |  |  |  |
| As | 0.125 | n.a. | 0.0188 | n.a. | 0.0147 |  |  |  |  |
| Ba | 0.0733 | n.a. | 0.0713 | n.a. | 0.0728 |  |  |  |  |
| Be | 0.00005 | n.a. | <0.00005 | n.a. | <0.00005 |  |  |  |  |
| B | 0.32 | n.a. | 0.33 | n.a. | 0.33 |  |  |  |  |
| Cd | 0.0001 | n.a. | <0.0001 | n.a. | <0.0001 |  |  |  |  |
| Ca | 52.3 | n.a. | 42.7 | n.a. | 45.6 |  |  |  |  |
| Cs | 0.003 | n.a. | 0.0048 | n.a. | 0.0047 |  |  |  |  |
| Cr | <0.0005 | n.a. | <0.0005 | n.a. | <0.0005 |  |  |  |  |
| Co | 0.00013 | n.a. | 0.0001 | n.a. | 0.00009 |  |  |  |  |
| Cu | <0.0005 | n.a. | <0.0005 | n.a. | <0.0005 |  |  |  |  |
| Fe | <0.02 | n.a. | <0.02 | n.a. | <0.02 |  |  |  |  |
| Pb | 0.0001 | n.a. | <0.0001 | n.a. | <0.0001 |  |  |  |  |
| Mg | 21.5 | n.a. | 18.6 | n.a. | 18.8 |  |  |  |  |
| Mn | <0.0005 | n.a. | <0.0005 | n.a. | 0.0009 |  |  |  |  |
| Mo | 0.006 | n.a. | 0.0083 | n.a. | 0.008 |  |  |  |  |
| Ni | <0.0006 | n.a. | 0.001 | n.a. | <0.0006 |  |  |  |  |
| K | 7.9 | n.a. | 9.8 | n.a. | 9.6 |  |  |  |  |
| Sc | 0.0001 | n.a. | 0.0001 | n.a. | 0.0001 |  |  |  |  |
| Se | 0.0004 | n.a. | 0.0002 | n.a. | 0.0003 |  |  |  |  |
| SiO_2_ | 23.7 | n.a. | 33.9 | n.a. | 34.1 |  |  |  |  |
| Ag | <0.00005 | n.a. | <0.00005 | n.a. | <0.00005 |  |  |  |  |
| Na | 69.1 | n.a. | 69.5 | n.a. | 69.3 |  |  |  |  |
| Te | <0.001 | n.a. | <0.001 | n.a. | <0.001 |  |  |  |  |
| Tl | 0.0002 | n.a. | 0.0002 | n.a. | 0.0002 |  |  |  |  |
| Th | <0.001 | n.a. | <0.001 | n.a. | <0.001 |  |  |  |  |
| Sn | <0.0001 | n.a. | <0.0001 | n.a. | <0.0001 |  |  |  |  |
| U | 0.0031 | n.a. | 0.0014 | n.a. | 0.0014 |  |  |  |  |
| V | 0.0011 | n.a. | 0.0014 | n.a. | 0.0011 |  |  |  |  |
| Zn | <0.002 | n.a. | <0.002 | n.a. | <0.002 |  |  |  |  |
| Cation-Anion Balance | -1.30% | n.a. | 0.70% | n.a. | 0.70% |  |  |  |  |
| Sum of Anions (meq/L) | 7.8 | n.a. | 6.9 | n.a. | 7.0 |  |  |  |  |
| Sum of Cations (meq/L) | 7.6 | n.a. | 7.0 | n.a. | 7.1 |  |  |  |  |
| Dissolved Nutrients (mg/L) |  |  |  |  |  |  |  |  |  |
| N as NO_3_ | 0.143 | 0.138 | 0.022 | 0.022 | 0.041 |  |  |  |  |
| N as NO_2_ | <0.002 | <0.002 | <0.002 | <0.002 | <0.002 |  |  |  |  |
| N as NH_3_ | 0.004 | 0.004 | 0.004 | 0.004 | 0.003 |  |  |  |  |
| P as O-PO_4_ | 0.003 | 0.003 | 0.003 | 0.003 | 0.004 |  |  |  |  |
| n.a. – Not applicable, sample not collected. | | | | | |  |  |  | n.a. |
