# Supplementary material for: A comparative study of prokaryotic diversity and physicochemical characteristics of Devils Hole and the Ash Meadows Fish Conservation Facility, a constructed analog
Source: PLoS One. 2018 Mar 15;13(3):e0194404. doi: 10.1371/journal.pone.0194404 (PMC5854365; doi:10.1371/journal.pone.0194404)
Supplement: S3 Table — (DOCX) [file pone.0194404.s005.docx]

**S3 Table. Cell counts (cells/mL) for planktonic samples collected at Devils Hole (DH), Ash Meadows Fish Conservation Facility (AMFCF), and Well P-9.**

|  | Sample | | | | |
| --- | --- | --- | --- | --- | --- |
| Replicate | DH Pool | DH Shelf | AMFCF Pool | AMFCF Shelf | Well P-9 |
| 1 | 62,500 | 66,667 | 104,167 | 75,000 | 183,333 |
| 2 | 75,000 | 87,500 | 83,333 | 91,667 | 162,500 |
| 3 | 100,000 | 87,500 | 87,500 | 75,000 | 191,667 |
|  |  |  |  |  |  |
| Mean (SD) | 79,167 (19,094) | 80,555 (12,028) | 91,667 (11,024) | 80,556 (9,623) | 179,167 (15,023) |
