# Supplementary material for: A comparative study of prokaryotic diversity and physicochemical characteristics of Devils Hole and the Ash Meadows Fish Conservation Facility, a constructed analog
Source: PLoS One. 2018 Mar 15;13(3):e0194404. doi: 10.1371/journal.pone.0194404 (PMC5854365; doi:10.1371/journal.pone.0194404)
Supplement: S4 Table — (DOCX) [file pone.0194404.s006.docx]

**S4 Table. Alpha diversity metrics for Devils Hole (DH), Ash Meadows Fish Conservation Facility (AMFCF), and Well P-9.**

|  |  | Alpha Diversity Metric | | | |
| --- | --- | --- | --- | --- | --- |
|  |  | OTU Richness | Chao1 | Faith's PD | Shannon Index |
| Devils Hole | Pool | 659.29 (8.50) | 941.91 (43.26) | 54.99 (0.72) | 5.97 (0.02) |
|  | Shelf | 101.50 (0.59) | 101.55 (0.74) | 14.96 (0.00) | 4.56 (0.02) |
|  | Sed1 | 614.48 (6.74) | 747.93 (24.68) | 45.46 (0.68) | 5.73 (0.02) |
|  | Sed2 | 774.79 (8.58) | 949.32 (32.97) | 56.10 (0.64) | 7.90 (0.02) |
|  | Sed3 | 651.04 (8.80) | 797.64 (29.24) | 47.85 (0.74) | 6.50 (0.03) |
|  | Sed4 | 885.77 (9.92) | 1046.08 (31.31) | 59.94 (0.81) | 8.07 (0.02) |
|  | Sed5 | 756.57 (7.02) | 904.22 (27.58) | 54.75 (0.60) | 7.51 (0.02) |
|  | Sed6 | 707.73 (9.19) | 868.93 (27.74) | 52.00 (0.67) | 7.01 (0.02) |
|  | Sed7 | 782.21 (9.26) | 966.29 (31.22) | 55.57 (0.78) | 6.87 (0.03) |
|  | Sed8 | 828.48 (10.74) | 1004.23 (31.10) | 57.00 (1.02) | 7.69 (0.03) |
| AMFCF | Pool | 333.80 (9.61) | 493.91 (44.51) | 34.62 (1.05) | 4.16 (0.03) |
|  | Shelf | 431.80 (10.06) | 622.90 (46.10) | 42.41 (0.99) | 4.96 (0.03) |
|  | Sed9 | 811.80 (8.35) | 964.29 (31.45) | 60.54 (0.66) | 7.85 (0.02) |
|  | Sed10 | 863.58 (10.33) | 1023.84 (28.55) | 62.92 (0.77) | 8.31 (0.02) |
|  | Sed11 | 756.22 (10.21) | 918.56 (35.71) | 58.20 (0.75) | 7.74 (0.02) |
|  | Sed12 | 752.87 (9.15) | 912.48 (29.92) | 58.58 (0.74) | 7.89 (0.02) |
|  | Sed13 | 888.54 (8.97) | 1036.99 (27.57) | 65.73 (0.69) | 8.35 (0.02) |
|  | Sed14 | 935.73 (10.73) | 1101.56 (32.30) | 66.57 (0.77) | 8.39 (0.02) |
|  | Sed15 | 705.72 (11.05) | 833.90 (29.41) | 52.55 (0.85) | 7.87 (0.02) |
|  | Sed16 | 812.63 (9.90) | 960.43 (27.54) | 59.96 (0.78) | 7.93 (0.02) |
| Well P-9 |  | 243.88 (3.54) | 274.58 (14.20) | 25.50 (0.35) | 6.67 (0.01) |
| Indices are presented as Mean (SD) based on 100 rarefactions of 10,000 sequences per sample. | | | | | |
