# Supplementary material for: A comparative study of prokaryotic diversity and physicochemical characteristics of Devils Hole and the Ash Meadows Fish Conservation Facility, a constructed analog
Source: PLoS One. 2018 Mar 15;13(3):e0194404. doi: 10.1371/journal.pone.0194404 (PMC5854365; doi:10.1371/journal.pone.0194404)
Supplement: S5 Table — (DOCX) [file pone.0194404.s007.docx]

**S5 Table. Results from Analysis of Similarity (ANOSIM) tests between planktonic communities in Devils Hole and AMFCF and between sediment communities in Devils Hole (DH) and AMFCF (Bray-Curtis, unweighted UniFrac, and weighted UniFrac dissimilarity calculated from an OTU table rarefied to 10,000 sequences per sample).**

|  | Bray-Curtis | Unweighted UniFrac | Weighted UniFrac |
| --- | --- | --- | --- |
| ^1^Planktonic Samples: DH vs. AMFCF | R=0.25, p=0.333 | R=0.5, p=0.333 | R=1, p=0.333 |
| ^2^Sediment Samples: DH vs. AMFCF | R=0.8599, p=0.001 | R=1, p=0.001 | R=0.8884, p=0.001 |
| ^1^24 permutations  ^2^999 permutations | | | |
