# Supplementary material for: A comparative study of prokaryotic diversity and physicochemical characteristics of Devils Hole and the Ash Meadows Fish Conservation Facility, a constructed analog
Source: PLoS One. 2018 Mar 15;13(3):e0194404. doi: 10.1371/journal.pone.0194404 (PMC5854365; doi:10.1371/journal.pone.0194404)
Supplement: S6 Table — Mean abundances and contributions to dissimilarity were calculated from an OTU table rarefied to 10,000 sequences per sample. (DOCX) [file pone.0194404.s008.docx]

**S6 Table. SIMPER analysis showing the top 5 OTUs responsible for the Bray-Curtis dissimilarity between planktonic communities in Devils Hole (DH) and AMFCF and between sediment communities in DH and AMFCF. Mean abundances and contributions to dissimilarity were calculated from an OTU table rarefied to 10,000 sequences per sample.**

| **Planktonic Samples: Devils Hole vs AMFCF** | | | | | | |
| --- | --- | --- | --- | --- | --- | --- |
| OTU ID | Phylum | Genus^1^ | Mean abund. DH (%) | Mean abund. AMFCF (%) | Contribution to dissimilarity (%) | Cumulative contribution to dissimilarity (%) |
| OTU_1850 | Verrucomicrobia | f_Verrucomicrobiaceae | 1.21 | 31.97 | 16.15 | 16.15 |
| OTU_502 | Cyanobacteria | unclassified genus | 16.64 | 0 | 8.74 | 24.89 |
| OTU_1062 | Proteobacteria (α) | f_Hyphomonadaceae | 0.01 | 13.15 | 6.90 | 31.79 |
| OTU_500 | Cyanobacteria | unclassified genus | 11.03 | 0 | 5.79 | 37.58 |
| OTU_941 | Planctomycetes | *Planctomyces* | 0 | 9.99 | 5.24 | 42.82 |
| **Sediment Samples: Devils Hole vs AMFCF** | | | | | | |
| OTU ID | Phylum | Genus^1^ | Mean abund. DH (%) | Mean abund. AMFCF (%) | Contribution to dissimilarity (%) | Cumulative contribution to dissimilarity (%) |
| OTU_500 | Cyanobacteria | unclassified genus | 13.81 | 0.0038 | 8.42 | 8.42 |
| OTU_2006 | Unassigned | unclassified genus | 4.21 | 2.54 | 2.46 | 10.88 |
| OTU_2023 | Unassigned | unclassified genus | 3.11 | 0.36 | 1.79 | 12.67 |
| OTU_162 | Bacteroidetes | f_Chitinophagaceae | 2.29 | 0.033 | 1.37 | 14.04 |
| OTU_291 | Chlorobi | o_PK329 | 1.95 | 0.12 | 1.12 | 15.16 |
| ^1^When genus was not the most specific taxonomy assignment for a particular OTU, the order (o_ prefix) or family (f_ prefix) is provided. | | | | | | |
